# Sonogenetics for precision medicine: from molecular toolkit to clinical translation

**DOI:** 10.7150/thno.134803

**Published:** 2026-06-04

**Authors:** Xinrui Yu, Min Pu, Ruoli Wang, Haitao Ran

**Affiliations:** 1 Department of Ultrasound, the Second Affiliated Hospital of Chongqing Medical University, Chongqing, 400010, China.; 2 Department of Neurosurgery, The Second Affiliated Hospital, Chongqing Medical University, Chongqing, China.

**Keywords:** sonogenetics, mechanosensitive ion channels, targeted gene delivery, microbubbles, precision medicine

## Abstract

Sonogenetics is an emerging technology for precise biological modulation. It utilizes ultrasound waves for remote, noninvasive, and spatiotemporally precise functional intervention in target cells genetically engineered for acoustic sensitivity. This review systematically summarizes the three core components comprising sonogenetics: sound-responsive agents for modulating cellular functions, highly efficient vectors for targeted gene delivery, and ultrasound emitters with optimized parameters. Furthermore, we delineate optimization strategies for these components and propose a standardized workflow for sonogenetic operations. This review proposes strategies to accelerate the transformation process of sonogenetics by analyzing current limitations in regulatory efficiency and clinical safety. Finally, this review discusses the existing technical challenges and transformation bottlenecks, and points out the promising directions of future research.

## 1. Introduction

Sonogenetics is a promising biomedical regulatory strategy that can be used to remotely control molecular and cellular activities in living organisms 1. This technology integrates ultrasound and genetic engineering, which can accurately regulate target cells. It can not only overcome the inherent depth limitations of optogenetics but also solve the problem of insufficient spatial resolution unique to systemic pharmacology. Sonogenetics shows immense promise in noninvasive treatment of diseases and the exploration of untouchable anatomical areas ranging from deep brain neural circuits to visceral tumors.

**Figure 1:**
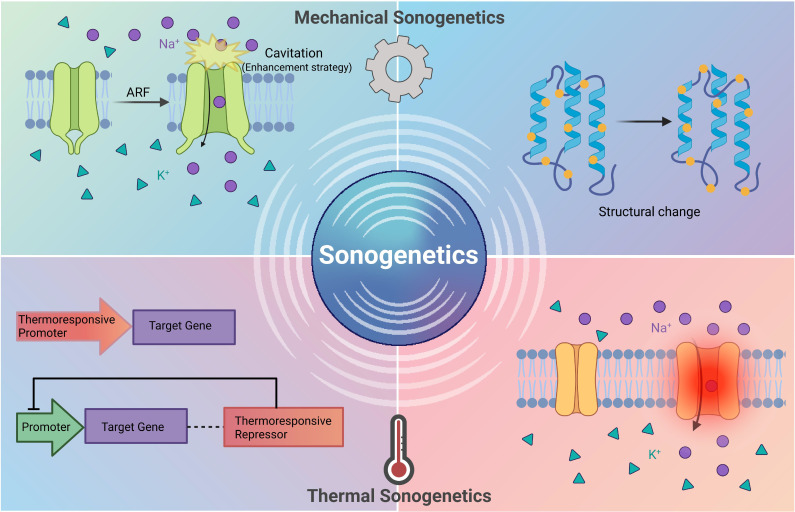
**Classification of sonogenetics.** Mechanical sonogenetics utilizes ARF and cavitation. Ultrasound directly triggers conformational changes of the exogenously expressed MS ion channels in target cells, producing a series of biological effects. Thermal sonogenetics uses thermal energy generated by ultrasound to activate thermoresponsive elements, such as heat-shock promoters or thermoresponsive repressors, to regulate gene expression. Created with BioRender.com. ARF: acoustic radiation force; MS: mechanosensitive.

According to their different physical mechanisms, sonogenetics can be divided into two categories 2-4 (**Figure 1**): mechanical sonogenetics and thermal sonogenetics. Mechanical sonogenetics mainly utilizes acoustic radiation forces (ARF) and cavitation effect of ultrasound 5. These mechanical forces directly trigger the conformational changes of the exogenously expressed mechanosensitive (MS) ion channels in target cells, thus regulating the excitability of cells 6. Thermal sonogenetics uses the thermal energy generated by ultrasound 7. This local temperature increase activates thermoresponsive elements, such as heat-shock promoters or thermoresponsive repressors, regulating gene expression 8. Although thermal sonogenetics strategies have practical value, their activation mechanisms and core design principles are different from those induced by mechanical effects. This review focuses on the strategies of mechanical sonogenetics.

The concept of "sonogenetics" was first proposed in 2015. With the help of microbubbles, researchers successfully activated the neurons that expressed TRP-4 ion channel of *Caenorhabditis elegans* by low-frequency ultrasound 9. This pioneering work not only lays the theoretical foundations for sonogenetics, but also demonstrates the potential of combining ultrasound with mechanosensitive media to accurately regulate the function of biomolecules. Since then, this field has entered a period of rapid development, and the scope of research has expanded from the activation of basic nerves in simple model organisms 9 to the regulation of complex physiological functions in mammalian models 10. The success of these studies shows that sonogenetics has developed into a noninvasive biological regulation tool with high spatiotemporal precision. A complete sonogenetics system usually includes the following tools: (1) an ultrasonic transmitter used to provide precise ultrasonic stimulation 11; (2) a sonosensitive mediator as the core of the technology; (3) an efficient and safe gene vector 12; (4) microbubbles to amplify ultrasonic mechanical forces 13,14, enhance gene delivery efficiency 15, open the blood-brain barrier (BBB) 16 and assist imaging 17, (5) reporter genes to track the expression site and efficiency of the target gene 18.

Although sonogenetics has made rapid progress, it still faces major obstacles to the translation from laboratory research to clinical technology. This review systematically outlines the core toolkit of mechanical sonogenetics and puts forward a clear optimization path. On this basis, we proposed a comprehensive technological workflow covering all aspects from imaging guidance to precise treatment. At the same time, this paper lists the main application areas of sonogenetics at present. This review compares sonogenetics with other precise modulation techniques, such as optogenetics and chemogenetics, in multiple dimensions to provide researchers with fresh research perspectives. Furthermore, this paper assesses the primary bottlenecks encountered in the clinical transformation of sonogenetics and proposes targeted solutions. With its continuous development, sonogenetics is expected to become a safe, efficient, and multifunctional precise treatment technology in clinical practice.

## 2. Mechanosensitive Transducers in Sonogenetics

**Figure 2:**
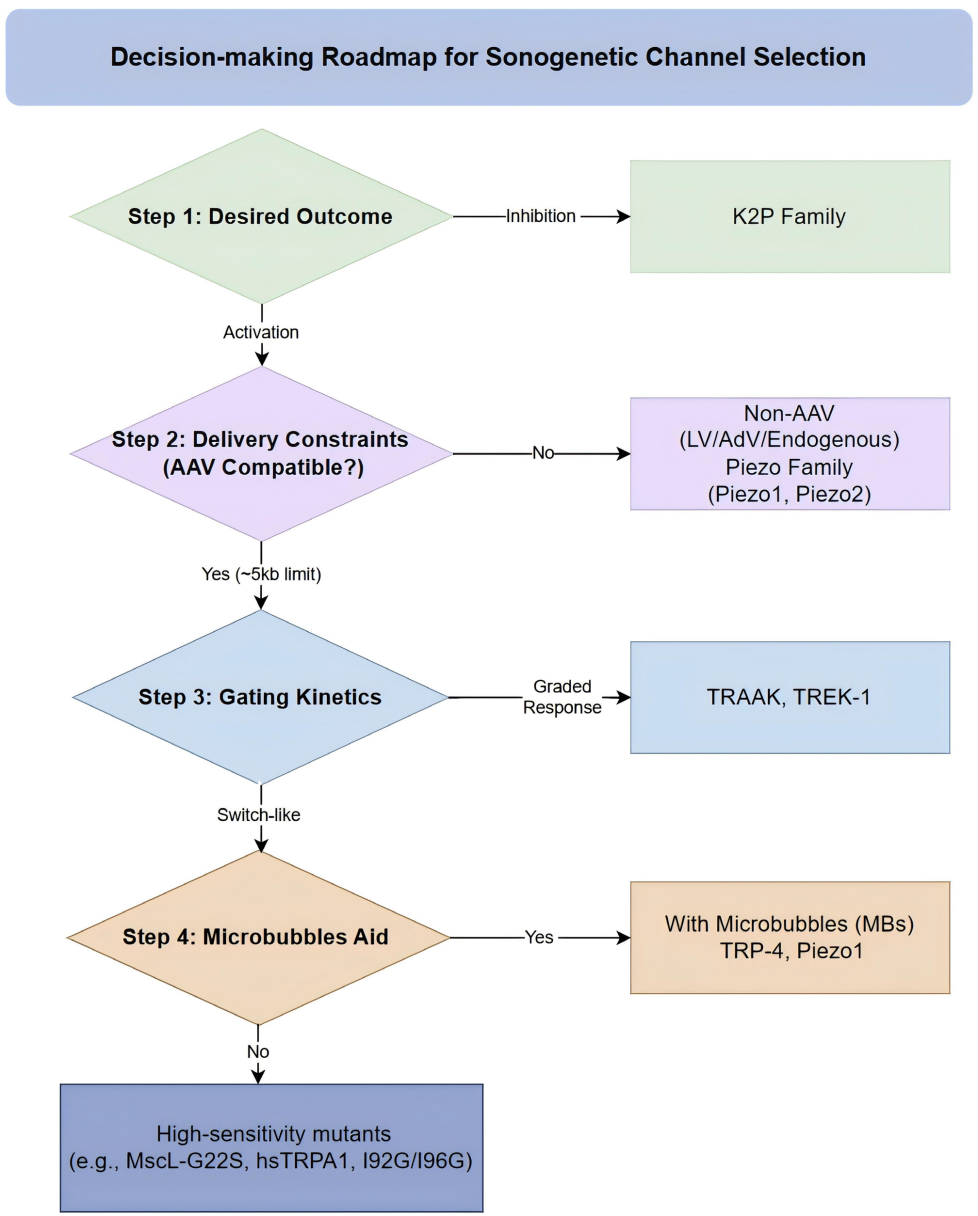
** A roadmap for the selection of sonogenetic channels.** This flowchart mainly divides the mechanosensitive transducers into four dimensions: desired outcome, delivery constraints, gating kinetics, and acoustic adjuvants. Based on this flowchart, researchers can make preliminary selections of mechanosensitive transducers according to the usage purpose and scenarios. AAV: adeno-associated virus; AdV: adenovirus; LV: lentivirus; MBs: microbubbles.

Mechanosensitive transducers are the mediators that convert ultrasound-induced mechanical stimuli into electrochemical signals (**Table 1**). The activation of these mechanosensitive transducers depends on two biophysical mechanisms: the bilayer model and the tether model 19,20. MS ion channels are common sonogenetic mediators. The ideal candidate is considered to have the characteristics of high acoustic sensitivity and expression without disrupting basal cellular physiology. By continuously optimizing mechanosensitive proteins, scientists have achieved a paradigm shift from microbubble-assisted activation to direct channel activation. To facilitate the selection of appropriate sonogenetic tools, we propose a strategic roadmap (**Figure 2**). The roadmap matches candidate channels with specific experimental goals. However, research results on activation thresholds, desensitization rates, and ion selectivity are often reported under different experimental conditions, and there is still a lack of a unified standardized comparison framework. The establishment of evaluation indicators is critical to promote sonogenetics from laboratory to clinical practice.

### 2.1 MscL in sonogenetics

The large-conductance mechanosensitive channel (MscL) was originally isolated from *Escherichia coli*. Its function is similar to an emergency release valve, which can protect bacteria from osmotic lysis 21. Structurally, MscL is composed of 5 identical subunits with a large pore diameter (25–30 Å) 22, offering a high conductance pathway for ions and small molecules. MscL has two transmembrane helices (TM1 and TM2) and an extracellular loop connecting two transmembrane regions. As the main part that forms the "pore" of the channel, the TM1 helix can alter channel behavior, such as sensitivity to mechanical stimulation 22-24.

The concept of "sonogenetics" was proposed in 2015. Yet as early as 2014, Heureaux *et al*. conducted pioneering research consistent with the core principles of sonogenetics. Heureaux *et al*. developed a tetracycline-regulatable adenoviral system to efficiently introduce wild-type (WT) MscL and its gain-of-function mutant MscL G22S into mammalian retinal pigment epithelial (RPE) cells. However, this approach required microbubble-mediated acoustic tweezing cytometry (ATC) rather than direct ultrasound activation 25. Subsequent studies have substantiated that some gain-of-function mutants could be activated by low-intensity focused ultrasound (LIFU) alone. The MscL-G22S mutant enabled region-selective neuromodulation in mouse brains via LIFU 12. Recent studies comparing MscL-G22S, MscL-G22N, and MS ion channels under identical conditions have further demonstrated the superior ultrasonic sensitivity of MscL-G22S 26. It has been demonstrated that the I92L mutant 27 and the I92G/I96G mutant are capable of lowering activation thresholds, with the latter exhibiting a significantly reduced activation threshold (0.044 MPa) compared to the I92L mutant and endogenous Piezo1 28,29. Although significant progress has been made in engineering MscL variants to optimize acoustic sensitivity, MscL's non-selective ion permeability poses risks of intracellular ion imbalance and cytotoxicity during long-term expression.

### 2.2 TRP in sonogenetics

Transient Receptor Potential (TRP) channels enable organisms to respond to various environmental stimuli. According to the homogeneity of amino acid sequences, the TRP channel superfamily is divided into seven subfamilies 30. They are involved in a range of other physiological and pathological conditions. As cationic channels, most members of the TRP superfamily can be activated by various stimuli. In addition to being mechanosensitive, TRP can respond to voltage, temperature, and ligands 31,32.

Initially, TRP-4 was identified as an essential pore-forming subunit of native mechanotransduction channels 33. Subsequently, ultrasound was demonstrated to affect TRP-4 MS ion channels expressed in *C. elegans* with the help of microbubbles to influence animal behavior 9,34. In mammalian systems, several TRP channels have been identified as responsive to ultrasound. Specifically, TRPP1/2 and TRPC1 are activated by ultrasound, leading to the influx of extracellular Ca²⁺, while the influence of cavitation and temperature has been ruled out 35. Moreover, the TRPM4 ion channel has been shown to act as a signal amplifier, enhancing the calcium accumulation signal triggered by the opening of MS channels 35. In the search for optimal sonogenetic tools, the human Transient Receptor Potential A1 (hsTRPA1) channel was demonstrated to exhibit superior responsiveness to ultrasound stimuli at 1 MHz, 2 MHz, and 7 MHz compared to other candidates such as MscL, Prestin, TRPV1, and Piezo1. The ultrasound sensitivity of hsTRPA1 likely depends on the structure of its N-terminal tip region and its interactions with the actin cytoskeleton and cholesterol 36. These findings provide a foundation for the subsequent design of mutants to improve sonogenetic efficiency. In endogenous contexts, TRPC6 has been proposed to act as a biosensor for ultrasound-induced mechanical distortions in the lipid bilayer of the mammalian brain. The activation is accompanied by sodium influx, resulting in the excitation of neuronal networks 37. While endogenous channels such as TRPP1/2, TRPC1, and TRPC6 enable "gene-free" modulation, they carry a risk of non-specific activation (off-target effects) in non-targeted tissues exposed to ultrasound. Furthermore, ultrasound neuromodulation via endogenous MS channels does not constitute true sonogenetics, as it lacks the genetic manipulation necessary for the precision of sonogenetics.

### 2.3 Piezo in sonogenetics

The Piezo family, including Piezo1 and Piezo2, is evolutionarily conserved 38. Piezo1 and Piezo2 possess a 38-transmembrane (TM) helix topology and form a homotrimeric propeller-shaped structure with a central ion-conducting pore and three peripheral mechanosensing blades. The curved TM region of three blades forms a nano-bowl configuration that can expand the in-plane membrane area, potentially conferring high mechanosensitivity to Piezo channels. 39,40. Piezo1 is activated by externally applied poking, stretching, and shear stress, or endogenously originating local membrane tension and myosin II-mediated traction force. In contrast, while Piezo2 is effectively activated by poking, it responds relatively poorly to stretch 41. Given the difference, Piezo1 is more widely used in sonogenetics.

The mechanosensitivity of Piezo1 has been harnessed for diverse therapeutic strategies. In cancer immunotherapy, ultrasound stimuli, assisted by microbubbles, activate endogenous Piezo1 in primary T cells (PBMCs) and Jurkat cells, resulting in a significant upregulation of anti-CD19 CAR (chimeric antigen receptor) gene expression 42. To further enhance specificity and reduce energy requirements, Piezo1-targeted microbubbles (PTMBs) were developed 43. Furthermore, in keloid treatment, 111 kHz low-frequency ultrasound was shown to inhibit fibroblast migration and increase total apoptosis of primary keloid fibroblasts (PKF) without the assistance of microbubbles by activating Piezo1 channels to trigger Ca²⁺ influx 44.

Because Piezo channels widely exist in multicellular organisms, future research may explore the differential expression of Piezo family channels across various tissues and their diverse functions within specific tissues when exposed to ultrasound. This is expected to further expand the application of ultrasonic regulation in biological functions and disease treatment. Moreover, as a typical large transmembrane protein 45, Piezo1 cannot be delivered efficiently through the mainstream AAV (adeno-associated virus) vectors. This limitation is mainly due to the fact that the size of its coding sequence exceeds the packaging capacity of AAV.

### 2.4 Prestin in sonogenetics

Prestin belongs to the SLC26 family of anion transporters and exhibits piezoelectric properties. It is different from the classical ion channels. It is a voltage-dependent anion transporter in mammalian cochlear outer hair cells. It converts changes in transmembrane voltage into cellular length alterations, thereby enabling high-frequency hearing and cochlear amplification 46-48. Furthermore, Prestin has also been observed to respond to ultrasound via ultrasonic-driven conformational transitions 49, generating electrical motion and functional modulation in cells.

Engineered Prestin (N7T, N308S mutant) was first utilized by Huang *et al*. as a sonogenetic tool to modulate mammalian cells and neurons while eliminating interference from the auditory pathway 50. Another study employed US-responsive DNA (Prestin plasmid, pPrestin)-loaded MBs (pPrestinMBs) to achieve noninvasive gene delivery by transiently disrupting the BBB, followed by ultrasound activation. Activation was observed in 58.2% of pPrestin-expressing cells, significantly higher than the activation rate in pPrestin-transfected cells without ultrasound activation 51. Recently, the Prestin (N548S, V715G) variant, enhancing prestin oligomerization, was developed through unbiased screening to further increase the efficacy of sonogenetic neurostimulation 52. These experiments suggested that ultrasound can effectively activate neurons expressing Prestin.

### 2.5 K2P in sonogenetics

Among the six two-pore domain potassium (K2P) subfamilies 53, only the TREK-1, TREK-2, and TRAAK channels within the TREK subfamily show mechanosensitivity 54. Mechanical force is transmitted directly to TRAAK and TREK via the lipid bilayer. Additionally, they also exhibit polymodal gating properties, responding to various physical and chemical stimuli such as arachidonic acid, temperature, pH, and lipids 55-57. Activation of mechanosensitive K^+^ channels leads to K^+^ efflux and subsequent hyperpolarization 58. This channel family is significant to sonogenetics, as it represents the first and currently only inhibitory toolkit in sonogenetics.

Sorum *et al*. developed a quantitative description of TRAAK, TREK-1, and TREK-2 activation by membrane tension and excluded the influence of thermal and cavitation effects. Among these, TRAAK exhibits the highest sensitivity. Furthermore, the result demonstrated that low-intensity and low-frequency ultrasound can increase the membrane tension and thus activate TRAAK channels. Notably, the study only quantitatively analyzed the tension-response curve and did not verify the activation effect of ultrasound on TREK-1 and TREK-2. Their work revealed two different gating modes: TRAAK and TREK-1 can provide a graded K^+^ conductance proportional to the size of mechanical force, whereas the activation of TREK-2 occurs in a narrower range of force values. This "switch-like" gate is similar to MscL and Piezo1 59-61. Another study demonstrated at the molecular level that ultrasound increases the current flowing through TRAAK, TREK-1, and TREK-2 channels, providing direct evidence for their ultrasonic responsiveness 29.

Hence, these channels have wide applications in sonogenetics. TRAAK and TREK-1 are suitable for applications that require graded and proportional modulation, such as delicate neuronal activity, while TREK-2 may be utilized for threshold-dependent activation. Verifying these inhibitory tools in mammalian models is still the first task to achieve bidirectional sonogenetic control. However, because some K2P channels, such as TRAAK and TREK-1 59,62, have endogenous expression, it is necessary to consider the off-target effects.

### 2.6 Potential candidates

In addition to currently known sonogenetic tools, the types of proteins that have been found to respond to ultrasound are still increasing. Recent studies have identified endogenous mechanotransducers with distinct ultrasound sensitivity, but their heterologous expression and application in sonogenetics remain underexplored. Investigating these candidates is significant for enhancing the precision and diversity of sonogenetic the toolkit.

In acid-sensing ion channels (ASICs), studies suggested that ASIC1a and ASIC3 can respond to ultrasound. ASIC1a is widely expressed in the brain. Lim *et al*. pointed out that ultrasonic stimuli can activate ASIC1a in the mouse brain through dynamic cytoskeletal rearrangement 63. Similarly, ASIC3 has been shown to play a role in mediating therapeutic ultrasound on chronic muscle pain 64.

Yes-associated protein (YAP) is a transcriptional mechanotransducer, not an ion channel. Ultrasound stimulation can reduce the phosphorylation level of YAP at Serine 127, thus driving its accumulation in the nucleus, and ultimately promoting the expression of proliferation-related genes 65. The study suggested that it has potential application value in regenerative medicine.

In touch receptor neurons, DEG/ENaC (degenerin/epithelial Na^+^ channel) protein plays a key role in mechanosensory transduction. Among these, the mec-4 gene is crucial to the mechanosensation of* C. elegans* 66. MEC-6, a component of the degenerin channel complex, is involved in mechanotransduction within touch cells 67. MEC-4 and MEC-6, expressed in the ALM and PLM neurons of *C. elegans,* have been demonstrated to jointly mediate neural activity and behavioral responses induced by ultrasound stimulation 68. Additionally, both TRP-4 and MEC-4 cooperate to transduce ultrasound stimuli into behavioral changes in *C. elegans* 34.

## 3. Gene Delivery Systems

**Figure 3:**
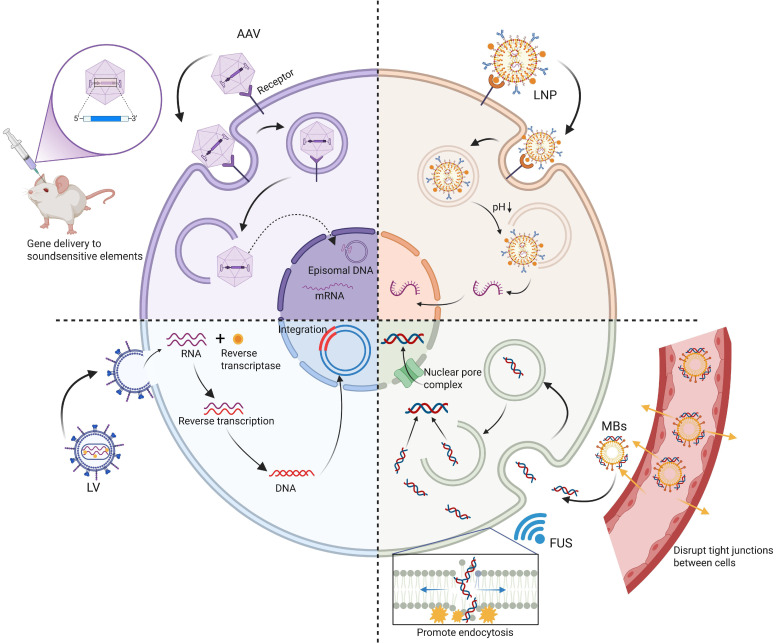
** The gene delivery system of sonogenetics.** It illustrates viral (AAV, LV) and non-viral (LNP, FUS combined with MBs) vectors used to deliver mechanosensitive protein genes to target cells. AAVs enter cells via endocytosis and are stably expressed in a free form in the nucleus; LVs utilize reverse transcriptase to integrate genes into the host genome to achieve permanent expression; LNPs, as a non-viral platform, can protect and release mRNA into the cytoplasm; the combination of FUS and MBs can enhance local gene delivery in specific regions to reduce the off-target effect through sonopororation effect and other mechanisms. Created with BioRender.com. AAV: adeno-associated virus; FUS: focused ultrasound; LNP: lipid nanoparticle; LV: lentivirus; MBs: microbubbles.

The efficient and safe delivery of MS channels is one of the key bottlenecks in the clinical translation of sonogenetics 70. An ideal gene delivery system must not only ensure successful expression of MS ion channels in the target tissue, but also balance factors such as expression duration, transgene packaging capacity, tissue targeting specificity, immunogenicity, and delivery efficiency of the delivery vehicle. The current strategies are mainly divided into two modes: viral vector and non-viral vector (**Figure 3**). In terms of sonogenetics, these two delivery methods have their own advantages and limitations (**Table 2**).

### 3.1 Viral vectors

Viral vectors leverage the intrinsic infectivity of viruses to transduce host cells. They protect their internal genetic material from the degradation of host enzymes. Due to the high gene transduction efficiency of viral vectors, they are currently the primary delivery tools of sonogenetics.

As ~25 nm icosahedral viruses without an envelope 71, adeno-associated viruses (AAVs) are widely employed in sonogenetics research because of their inherent safety and high biocompatibility 72,73. AAV genomes can achieve long-term transgene expression for several years as non-integrated episomes in the nucleus 74. However, because the genes introduced by AAV are in a free state, the risk of their loss in the process of cell division is high 75. Specific serotypes (e.g., AAV9) and engineered variants (e.g., AAV9.7m8) can accurately target the central nervous system (CNS) 76 and primary visual cortex (V1) 6. The use of small-size cell-specific promoters like human synapsin (hSyn) or CaMKII further enhances specificity 6. The delivery strategy has been verified in many sonogenetic studies, including the precise expression of MscL-G22S in primary neurons 12 and the expression of Cre-dependent TRPA1 in cortical neurons 36. Despite these advantages, the application of AAVs is still subject to the inherent limitation of limited packaging capacity (~5 kb) 77. This often forces researchers to strike a trade-off between functionality and specificity. This is a primary obstacle that hinders the wide application of AAVs in sonogenetics.

Lentiviral vectors (LVs) have larger packaging capacity (~9 kb) 78 and the ability to integrate into the genome. However, for *in vivo* applications, people are still concerned about insertional mutagenesis and immunogenicity. LVs have been employed to stably express MS channels such as TRPV1 35 and MscL 28 in primary neurons and cancer models. The development of doxycycline-inducible (Tet-on) lentiviral system facilitates precise, temporally controllable expression of MscL 28. Additionally, pseudotyping technology enhances the targeting efficiency of LVs. This is achieved by replacing envelope proteins to modulate the receptor-binding specificity 80. Examples include using rabies virus glycoproteins to target neurons 81 or using VSV-G fused with anti-EGFR scFv pseudotype to target tumors 82.

Similar to AAVs, adenoviruses (AdVs) deliver genetic material without genomic integration 83. AdVs can carry more than 30 kb of exogenous DNA, which is far more capacity than AAVs and LVs. However, their strong immunogenicity severely restricts their *in vivo* application 84. Nevertheless, AdVs are still of great value in specific experimental situations. Heureaux *et al*. successfully utilized the recombinant AdV system to achieve high-efficiency, dose-dependent expression of MscL in retinal pigment epithelial cells 25.

### 3.2 Non-viral vectors

To avoid the inherent immunogenicity and production costs of virus vectors, the non-viral vectors have become an attractive alternative. Non-viral vectors will not be integrated into the genome of host cells, which eliminates the risk of insertion mutations and improves the safety of gene delivery. Moreover, these vectors can accommodate larger transgenes and are suitable for mass production. The two major challenges of non-viral gene delivery are the relatively low transfection efficiency and the typically transient expression. However, the short duration of transgenic expression is not an absolute disadvantage, depending on the treatment goal.

As a multifunctional, low-immunogenicity non-viral platform 85, lipid nanoparticles (LNPs) are used in sonogenetics 86. By modifying their compositions, they can target specific cell types or change their metabolic processes. Their transient expression is suitable for short-term sonogenetic interventions, which helps to reduce the potential cytotoxicity caused by the long-term accumulation of exogenous MS channels. There has been a study using cationic nanoliposomes to deliver *MscL I92L* plasmids to tumor cells 87. Despite these advantages, the *in vivo* application of lipid nanoparticles (LNPs) is still limited by their inherent liver accumulation effect and limited transfection efficiency.

Microbubble-assisted ultrasound-guided gene delivery is regarded as a promising gene delivery method, which is especially suitable for applications in the CNS 88. Utilizing focused ultrasound (FUS) to trigger microbubble cavitation, this method can not only be used as an adjuvant to transiently overcome natural barriers, such as the BBB 89,90, but also directly act as a gene carrier 91. This ensures that gene entry is strictly limited to the irradiated focal area, thus minimizing the off-target effect 92. This approach has been successfully verified in the mouse brain. In the study, 1-MHz FUS and plasmid-loaded microbubbles were used to target the BBB and deliver the* Prestin* gene to specific neurons 51. Despite its potential, the clinical translation necessitates the precise optimization of gene-loading capacity for large sequences 91, microbubble stability, and ultrasonic parameters 93.

## 4. Optimization Strategies for Sonogenetics

**Figure 4:**
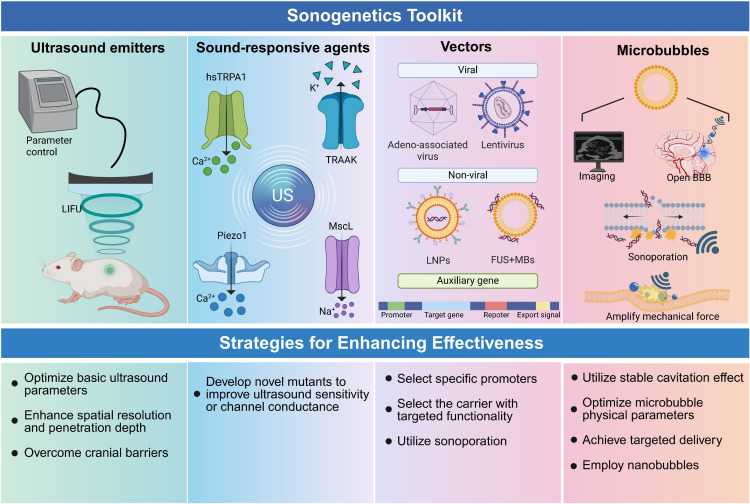
** Sonogenetics toolkit and strategic pathways for enhancement.** (1) Summarizes the essential components, including ultrasound emitters for parameter control, sound-responsive agents (e.g., hsTRPA1, Piezo1), viral and non-viral vectors for gene delivery, and microbubbles as amplifiers. (2) Outlines key optimization pathways to improve efficiency and safety regarding the aforementioned four aspects. Created with BioRender.com. BBB: blood-brain barrier; LIFU: low-intensity focused ultrasound; US: ultrasound.

The clinical translation of sonogenetics hinges on enhancing its efficiency, safety, and precision. To achieve these objectives, we propose optimization strategies primarily focused on three dimensions: ultrasound stimulation parameters, mechanosensitive proteins, and microbubble adjuvants (**Figure 4**).

### 4.1 Gas-filled microbubbles and nanobubbles

Gas-filled bubbles serve as amplifiers in sonogenetics by leveraging stable cavitation. These amplified forces facilitate the activation of MS ion channels in cell membranes at lower ultrasound energy. The modifiability of gas-filled bubbles allows for further system optimization. However, experiments need to avoid inertial cavitation that is induced by bubble rupture, resulting in tissue damage. Notably, if a system lacks genetic manipulation steps, regulation assisted solely by microbubbles does not constitute sonogenetics.

Microbubbles are the most commonly used sonoadjuvants. In *C. elegans,* MBs facilitate behavioral responses at low acoustic pressures (0.79 MPa). In contrast, the response differential between presence and absence of MBs diminishes at high pressures, as the high-pressure ultrasound alone provides enough mechanical force for activation. This confirms their role in lowering the energy barrier required for stimulation 34. The amplification effect is size-dependent, with smaller MBs (1.5–2.5 μm) triggering stronger responses than larger variants (4–6.5 μm) 9. Furthermore, PTMBs have been developed to enhance targeting specificity. This molecular targeting technology enables cell-specific activation and seven adhered MBs induce a stronger response than three 43. Optimizing the number, size, and targeting ability of MBs is essential for advancing sonogenetic efficacy.

With the advancement in nanotechnology, gas-filled nanostructures have been used to enhance safety, precision, and efficacy of ultrasound-mediated neuromodulation. Hou *et al*. utilized gas-filled nanostructures, gas vesicles (GVs), to enhance ultrasound sensitivity. Under ultrasound pressure (1 MHz, 0.28 MPa), only harmonic signals were observed, indicating that GVs did not induce the collapse of gas bubbles to damage tissue. This result confirms that nanostructures have better safety 107. Recently, PEGylated gas vesicles (PGVs) were developed with FDA-approved polymer shells to improve biocompatibility. These nanostructures facilitated the precise modulation of deep-brain circuits with extremely high spatial resolution. This method can distinguish brain regions only ~1.1 mm apart, and selectively induce different behaviors such as "rotation" and "freezing" 10. Compared with microbubbles, nanobubbles are easier to penetrate the tissue gaps, achieving noninvasive targeted delivery and supporting high-precision spatial neuromodulation.

### 4.2 Ultrasonic equipment and ultrasound parameters

The ultrasound stimulation device controls the key acoustic parameters that affect sonogenetic results, including frequency, intensity, pulse duration, and waveform 108, by modulating mechanical forces, thermal effects, and cavitation effects 109. Ideally, the stimulation device should provide flexible parameter optimization to adapt to the kinetic properties of target MS channels. Finding the balance is essential to improve activation efficiency and reduce risks, including desensitization, excessive thermal deposition, or inertial cavitation.

Currently, there is a lack of unified standards for ultrasound parameters in this field. Sound pressure is usually the direct driving force that turns on the MS channel 12. The choice of frequency usually depends on the required depth of tissue penetration, which must be balanced with spatial precision. Deep tissue requires low-frequency ultrasound to ensure sufficient penetration 11. In the study of visual restoration, the selection of frequency also needs to consider the repetition rates required for visual recovery 6.

Once channels are activated and achieve the expected effect, the focus should be turned to safety. When employing MBs, researchers should pay attention to the Mechanical Index (MI) to ensure that operation is carried out within a stable cavitation window 110. The lower duty cycle (DC) emphasizes the mechanical effects of LIFU, which can improve the safety of sonogenetics. Intensity is defined as power per unit area. The total energy delivery is quantified through spatial peak pulse average intensity (I_SPPA_) and spatial peak temporal average intensity (I_SPTA_). The current ultrasound safety regulations are based on FDA diagnostic guidelines. It is stipulated that I_SPPA_ should be less than 190 W/cm^2^, and I_SPTA_ should be less than 720 mW/cm^2^, and MI should be less than 1.9 111. However, the safety parameters of therapeutic ultrasound still need to be further studied.

CNS regulation is an important application field of sonogenetics, and the development of related equipment can significantly enhance therapeutic efficacy. The cranium causes significant signal attenuation and distortion 112,113. To solve this problem, time-shifting techniques within phased array transducers are used. They can control all waves to reach the target at the same time, so as to correct distortions 114. For cortical regions, high-frequency ultrasound (15 MHz) can be transmitted through cranial windows implanted in mice to achieve precise stimulation of the V1 cortex 6. In addition, a miniature wearable ultrasound system integrating a self-focusing transducer and bioadhesive hydrogel has been developed for long-term neuromodulation. This ultrasonic transmission can not only pass through the 8 mm human skull, but also achieve a smaller focal spot size and higher spatial resolution 115. Equipment with high spatial resolution and portability is the key direction for the future development of sonogenetic hardware.

The collaborative integration of multiple enhancement strategies usually brings better results. Although LIFU has significant advantages in penetrating bones and tissues, its spatial resolution is inevitably affected by its longer wavelengths 11. Combining low-frequency ultrasound with target-modified bubbles can theoretically resolve the trade-off. This combination may significantly enhance the interaction between ultrasound and tissue in the MS ion channel of the targeted cells by these target-modified bubbles, so that deep-tissue cells can be accurately activated with high spatial resolution. Such collaborative approaches are vital to give full play to the therapeutic potential of sonogenetics.

### 4.3 Ion channel mutants

MS ion channels are the core of sonogenetics. Some wild-type MS channels are naturally insensitive to LIFU, which limits the efficiency of sonogenetics. By engineering novel channel mutants with enhanced ultrasound sensitivity or conductance, safer, lower-pressure ultrasound can be used to elicit equivalent biological responses.

Mutants of the MscL channel represent the most thoroughly studied sonogenetic tools. The hydrophobicity of G22 determines pressure sensitivity of the MscL channel. The replacement of this residue with a hydrophilic residue can reduce the gating threshold 116. The MscL-G22N mutant (Gly-to-Asn) damaged the hydrophobic "lock", reducing the pressure required for channel opening to below 5 mmHg 117. In hypo-osmotic solution, the MscL-G22S mutant (Gly-to-Ser) expressed in retinal pigment epithelium (RPE) showed better activation effect than WT MscL 6. The MscL-I92G/I96G double mutant was engineered to modify mechanosensitivity through TM1-TM2 interaction 118. Its activation requires ultrasound pressure of only 0.044 MPa (26% Amplitude), which is significantly lower than the 0.053 MPa (31% Amplitude) required by WT MscL 28.

In addition to the mutants of MscL, analysis of Prestin protein sequence among non-echolocating and echolocating species revealed that the 7th and 308th amino acids often switch from N to T and from N to S, respectively. These observations led to the generation of Prestin mutants: Prestin (N7T), Prestin (N308S), and Prestin (N7T, N308S). The ultrasound sensitivity of mammalian cells expressing Prestin (N7T, N308S) was ~11-fold higher than that of control cells under low-frequency (0.5 MHz), low-energy (0.5 MPa and 0.1% of duty cycle), and transient (3 s) ultrasound conditions 50,51. Future research should clarify the mechanisms of action of existing highly sonosensitive channels, explore novel mutant libraries to further optimize ultrasound responsiveness, and screen out new ultrasonic response candidate molecules from a variety of organisms.

## 5. Visualization

**Figure 5:**
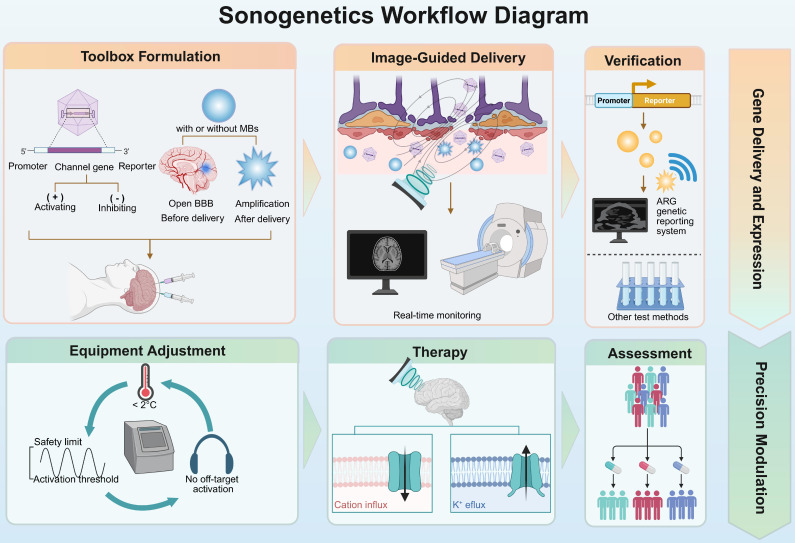
** Schematic diagram of the workflow of sonogenetics.** The process is divided into two primary phases. The first stage is gene delivery and expression, including vector formulation, image-guided delivery, and verification; the second stage is precision modulation, which involves parameter optimization, therapeutic stimulation of MS channels, and efficacy assessment. Created with BioRender.com. ARG: acoustic reporter gene; BBB: blood-brain barrier; MBs: microbubbles; MS: mechanosensitive.

The lack of real-time visibility in the process of *in vivo* gene delivery poses significant safety concerns and hinders clinical translation. Visualization strategies aim to establish a closed-loop framework covering "precise delivery, real-time monitoring, and efficacy verification." This framework is designed to assess whether the genes have reached the target and been successfully expressed. This can meet the clinical demand for noninvasive and quantifiable treatment evaluation. Real-time imaging monitoring of gene delivery and reporter gene expression is the basis for providing safety and precision. Consequently, we propose a sonogenetic workflow that integrates visualization techniques, which makes sonogenetics have preliminary clinical feasibility (**Figure 5**).

### 5.1 Image-guided gene delivery

Currently, imaging-guided gene delivery strategies have not been widely applied in basic sonogenetics. The delivery primarily relies on stereotactic or local injections to ensure that the gene vectors reach the target site directly. However, in view of the requirements of clinical translation for noninvasive precision, image-guided strategy has become a priority.

The following noninvasive monitoring methods hold promise for applications in sonogenetics. Magnetic resonance-guided focused ultrasound surgery (MRgFUS) is a pivotal technology: the three-dimensional imaging feature of MRI provides precise spatial localization for ultrasound focus, while FUS transiently opens the BBB to enhance vector concentration at the target site 119. Human clinical trials delivering therapeutics have demonstrated there are no serious treatment-related side effects 120. In nonhuman primates (NHPs) and humans, the technology has been used to facilitate the entry of AAV vectors carrying a green fluorescent protein (GFP) reporter gene and the hSyn promoter into the brain, yielding a 7–50-fold increase in GFP-positive neurons in the BBB-opened region compared to unopened regions 121. Similarly, ultrasound has been used to guide the AAV9-GFP delivery during *in utero* fetal gene therapy of pigs. The turbulent flow in fetuses was observed to ensure precise amniotic cavity injection and avoid off-target effects 122. Ultrasound and MB-guided adenoassociated viral vector (UMGAAV) utilizes microbubbles as real-time indicators of AAVs. The "blooming" artifacts induced by MB destruction confirm the treatment area, while increasing vector internalization 123.

### 5.2 Genetic reporting system

Reporter genes are introduced to indicate successful expression following delivery. These genes are co-delivered and co-expressed in the host cells together with the target gene. The expression of the reporter gene can reflect the activity of the target gene, the localization of the target protein, and the overall transduction efficiency.

Fluorescent reporter genes have become important tools for *in vitro* mechanism validation due to their intuitive visualization and high spatial and temporal resolution. After incident light that excites the wavelength reaches the fluorophore in the cell, the fluorescence will release photons of a distinct emission wavelength and be detected by a specialized camera 124,125. Green fluorescent proteins (GFP), mCherry, and yellow fluorescent protein (YFP) are often used as reporters. In sonogenetic studies, GFP variants have been employed to visualize the expression of MscL-G22S in HEK293T cells 12 and Prestin constructs in primary neurons and brain slices 51. Similarly, red fluorescent proteins like mCherry have been utilized to verify the successful expression of MscL mutants 28. However, the application of fluorescent reporter genes in *in vivo* experiments is limited by their poor light penetration and background fluorescence interference. Other optical reporter genes also face similar problems. Hence, these reporter genes can only be used for surface tissue or *in vitro* analyses 126.

In contrast, acoustic reporter genes (ARGs) offer the advantages of deep tissue penetration, low background signal, and accurate localization, making them ideal for *in vivo* applications. ARGs are based on GVs. GVs are air-filled protein nanostructures found in certain waterborne bacteria and archaea. Their ability to scatter sound waves lays the foundation for them as reporter genes. In 2018, Bourdeau *et al*. first developed ARGs for noninvasive ultrasound imaging of intra-mammalian microbiomes based on genetically engineered GVs 127. In 2019, ultrasound imaging of gene expression was successfully achieved in mammalian cells by engineering gene clusters to adapt to the transcription and translation systems of mammalian cells. Ultrasound images clearly revealed the localization of mARG-expressing cells. Calculating the signal difference between pre-collapse and post-collapse of GVs can eliminate background scattering from host tissues, which clearly confirms that the ultrasound signals originate from mARG-expressing cells 128. Technical optimizations can further improve the imaging quality. The burst ultrasound reconstructed with signal templates (BURST) increased the sensitivity of ARGs ~1,000-fold using two pressure level ultrasound 129. mARG_Ana, an improved mammalian ARGs, produces 38-fold stronger nonlinear contrast than first-generation versions. By applying the xAM pulse sequence, nonlinear signals are enhanced while linear background scattering is canceled out from host tissues. Unlike BURST, this method does not rupture GVs, enabling non-destructive imaging 130.

Notably, GVs are multifunctional, facilitating Theranostics for sonogenetics. Beyond imaging gene expression, GVs can also amplify mechanical forces through stable cavitation to enhance channel activation. Furthermore, the inertial cavitation effect induced by high-pressure ultrasound can produce therapeutic effects, such as targeted destruction of tumor tissues. These functions make ARGs a promising choice to replace optical reporters in noninvasive, deep-tissue imaging in sonogenetics.

## 6. Applications of Sonogenetics

Sonogenetics harnesses the unique synergy between the deep tissue penetration of ultrasound and gene-targeted cell specificity, and is emerging as a transformative noninvasive biomedical intervention. This section elaborates on the expanding transformation research prospects of sonogenetics, highlighting its core roles in three key areas, including the treatment of neurodegenerative and neuropsychiatric disorders through precise regulation of neural circuits, noninvasive restoration of sensory function, and the induction of anti-tumor reactions under spatiotemporally controllable conditions (**Table 3**). These applications together show the potential of sonogenetics in reshaping precision medicine.

### 6.1 Neuromodulation

Neuromodulation aims to precisely regulate neural activity, which is crucial for the study of brain function and the treatment of functional disorders. Currently, the main methods of neural regulation include: deep brain stimulation (DBS), optogenetic interventions, transcranial magnetic stimulation (TMS), and transcranial direct current stimulation (tDCS). However, these techniques often have problems such as limited spatial resolution and penetration depth, or immune responses caused by invasiveness, which further restricts the application of some technologies 131. Therefore, there is an urgent need for a noninvasive neuromodulation technology with high spatiotemporal accuracy and deep tissue penetration ability. Sonogenetics overcomes these limitations by using FUS to achieve deep tissue penetration and the use of mechanosensitive transducers that exhibit responsiveness to FUS 27.

**Figure 6:**
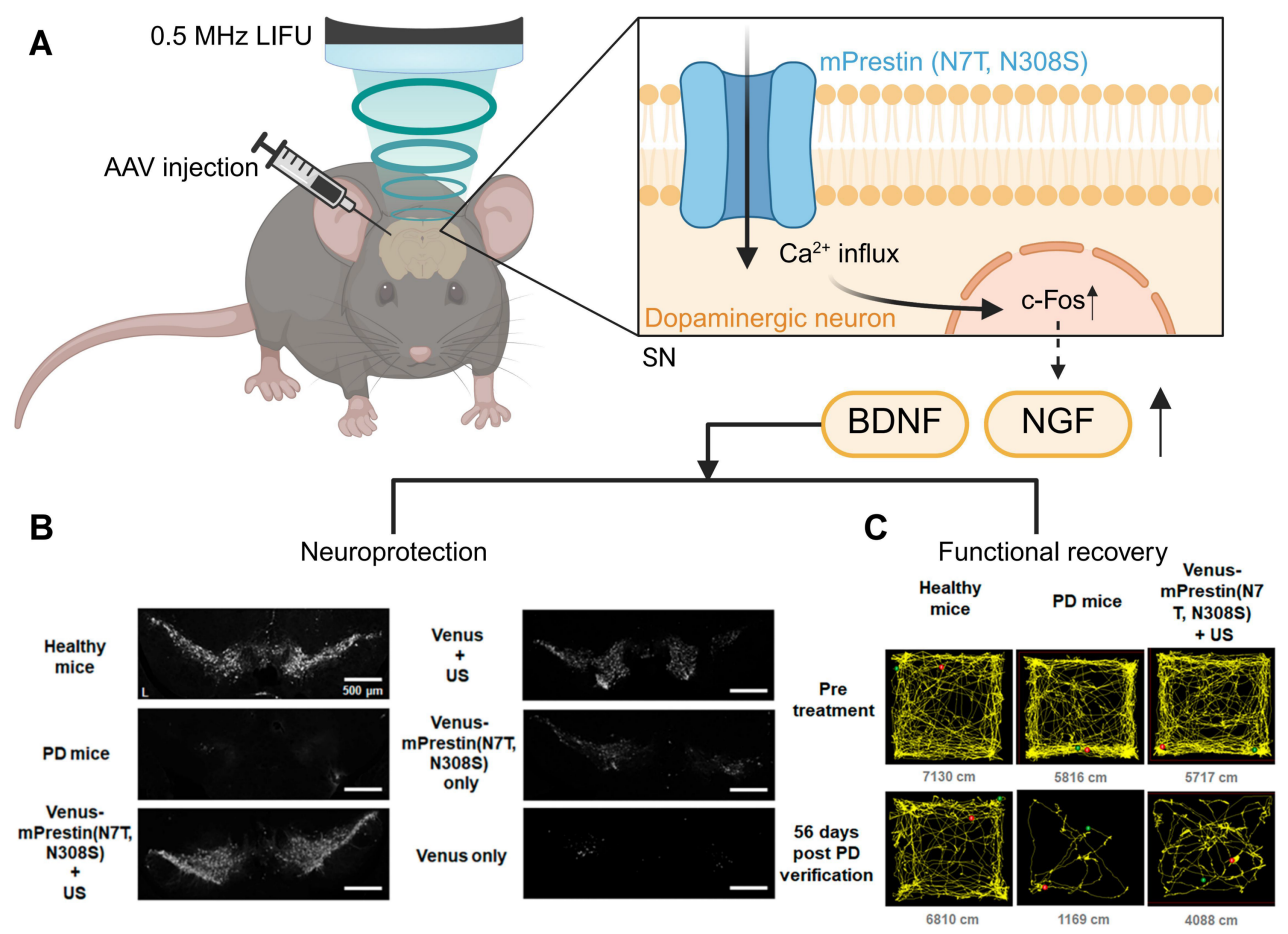
**Schematic illustration of sonogenetic-based neuromodulation for the amelioration of Parkinson's disease. (A)** The process of sonogenetics treats for Parkinson's disease.** (B, C)** This sonogenetic treatment results in neuroprotection, evidenced by preserved tyrosine hydroxylase-positive (TH⁺) neurons, and functional recovery, demonstrated by improved locomotor activity in open field tests. Adapted with permission from 132, Copyright 2021 American Chemical Society. Recreated with BioRender.com. AAV: adeno-associated virus; BDNF: brain-derived neurotrophic factor; c-Fos: cellular FBJ murine osteosarcoma viral oncogene homolog; LIFU: low-intensity focused ultrasound; NGF: nerve growth factor; PD: Parkinson’s disease; SN: substantia nigra; TH: tyrosine hydroxylase; US: ultrasound.

Sonogenetics can stimulate the deep brain without permanent implantation. It has a unique advantage in the treatment of neurodegenerative diseases. Parkinson's disease (PD) has motor and non-motor symptoms due to the degeneration of dopaminergic neurons 133. Current treatment mainly focuses on symptom management. However, preclinical studies using sonogenetics have shown that this technology is expected to slow down the progression of the disease. In the Parkinson's disease mouse model, MscL-G22S was expressed in the subthalamic nuclei. Ultrasound-stimulated MscL-PD mice improved their balance and motor endurance through the modulation of specific brain circuits 134. The dopaminergic neurons of the substantia nigra in PD mice expressed mPrestin (N7T, N308S) (**Figure 6A**). After ultrasound stimulation, the loss of TH-positive neurons in the substantia nigra (SN) significantly slowed down. In addition, repeated neuronal stimulation upregulated the expression of brain-derived neurotrophic factor (BDNF) and nerve growth factor (NGF), promoting neuroprotection and synaptic plasticity. Consequently, a significant enhancement of the motor function of PD mice was observed (**Figure 6B, C**) 132.

Epilepsy is a chronic brain dysfunction caused by abnormal synchronous excitation of neurons in the brain, manifested as convulsions and disturbances of consciousness. The present research generally has problems such as high invasiveness, insufficient targeting accuracy, and off-target effects 135-137. Studies demonstrated that GABAergic neurons in the amygdala in the deep brain can inhibit neuronal excitation by specifically releasing gamma-aminobutyric acid (GABA), which is crucial for regulating epileptic seizures 138. A study employing sonogenetics to treat epilepsy precisely harnessed this mechanism by expressing mPrestin (N7T, N308S) in GABAergic neurons. These neurons were activated via 0.5 MHz transcranial ultrasound, causing the release of GABA. GABA inhibited the abnormal synchronous excitation of nerve cells. In a rat epilepsy model induced by pentylenetetrazole, ultrasound stimulation directly reduced epileptiform abnormal activity by 50%, with the inhibitory effect persisting for 60 minutes. This study effectively suppresses epileptic seizures by ultrasonic remote control of GABA release 139. Furthermore, sonogenetics plays a crucial role in modulating the reward circuitry of the ventral tegmental area (VTA). Under ultrasound stimulation, VTA neurons expressing MscL-G22S induced a rapid synchronous increase in DA signaling in the nucleus accumbens (NAc), thus regulating appetitive conditioning. In addition, the dorsal striatum (dSTR) expressing MscL-G22S can respond to ultrasound and increase locomotion in freely moving mice 134. These studies demonstrate that sonogenetics has unique potential compared with traditional cerebral stimulation techniques.

In the research of endogenous sonosensitive mediators, PGVs+US stimulation in the dorsal raphe nucleus (DRN) was demonstrated to induce rapid release of 5-HT with exceptional temporal precision, effectively alleviating depression in mouse models 10. While sonogenetic neuromodulation relying on engineered and exogenously expressed channels is undoubtedly important, the strategic harnessing of endogenous sonosensitive mediators within the cranium, such as Piezo1 in neurons 140 and TRPA1 in astrocytes 69, can eliminate dependence on gene delivery.

### 6.2 Vision restoration

Visual impairment severely compromises quality of life. Beyond conventional aids, gene therapies 141 and optogenetics have been demonstrated at the clinical level 142. However, optogenetics is fundamentally limited by significant absorption and light scattering in brain tissue 143. Sonogenetics takes the non-contact penetrative advantage of ultrasound to enable deep and long-range activation across the cortex. This approach is less invasive than current brain-machine interface (BMI) methods employed for visual restoration.

**Figure 7:**
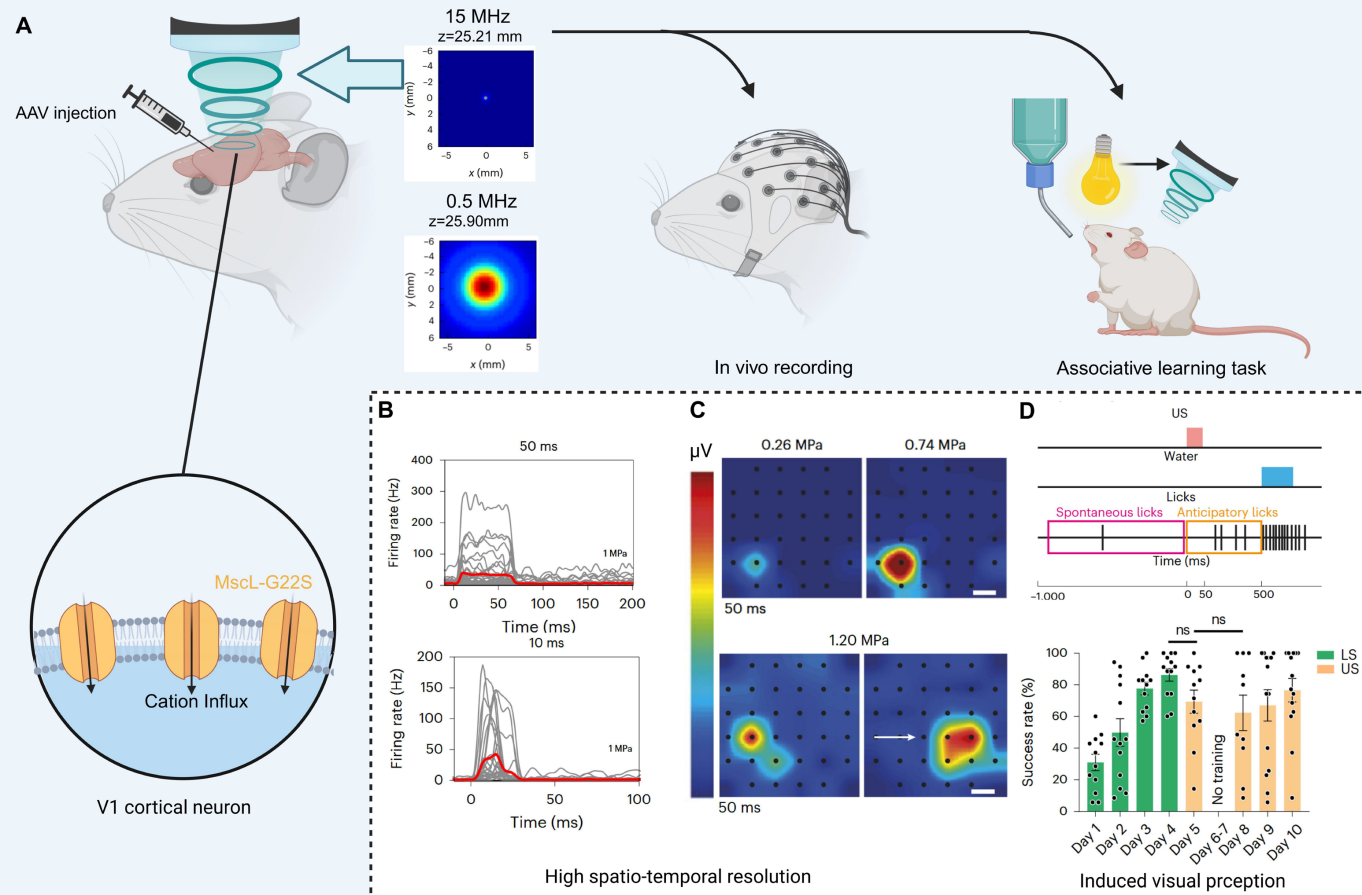
** High spatiotemporal resolution sonogenetic therapy for visual restoration using the MscL-G22S channel. (A)** The treatment and experimental procedures for vision restoration induced by sonogenetics and comparison of acoustic beam profiles. **(B)** SDF shows millisecond-scale temporal precision in response to 10–50 ms pulses. **(C)** Pseudocolor activation maps reveal sub-millimeter spatial confinement that shifts precisely with transducer displacement. **(D)** In an associative learning task, mice expressing MscL-G22S in V1 perceive ultrasound stimulation as a visual cue. Adapted with permission from 6, copyright 2023 Springer Nature. Recreated with BioRender.com. AAV: adeno-associated virus; SDF: spike density function; V1: primary visual cortex.

High spatiotemporal resolution is crucial for visual recovery to match visual frame rate and generate high-quality visual images. Toward this goal, Cadoni *et al*. demonstrated in both cellular and animal studies that sonogenetics provides the resolution necessary for vision restoration (**Figure 7B, C**). They chose the AAV to deliver MscL-G22S (**Figure 7A**). 15 MHz ultrasound stimulation evokes responses in the V1 cortex with the neural response latency under 10 ms. Moreover, the responses of the V1 cortex follow a repetition rate of up to 13 Hz that aligns with the transmission frequency required for visual recovery 144. The spatial resolution of the V1 cortex is approximately 400 µm, compatible with the parameters required for visual restoration. Behavioral experiments further confirmed that 15 MHz ultrasonic stimuli can be perceived as visual by mice expressing MscL (**Figure 7D**). However, generating useful vision will necessitate sophisticated spatiotemporal patterning of ultrasound stimuli to construct coherent images, presenting a significant neural coding obstacle 6. Whether the mechanical forces generated during treatment could damage the V1 cortex, and how ultrasound can reach the V1 cortex in human applications, are also issues that require attention.

### 6.3 Tumor therapy

Conventional cancer therapies like chemotherapy and radiotherapy lack sufficient precision, leading to systemic side effects 145. Sonogenetics offers a novel strategy by integrating the deep penetration of focused ultrasound with genetically engineered mechanosensitivity. This approach triggers tumor-specific apoptosis while sparing healthy surrounding tissue.

**Figure 8:**
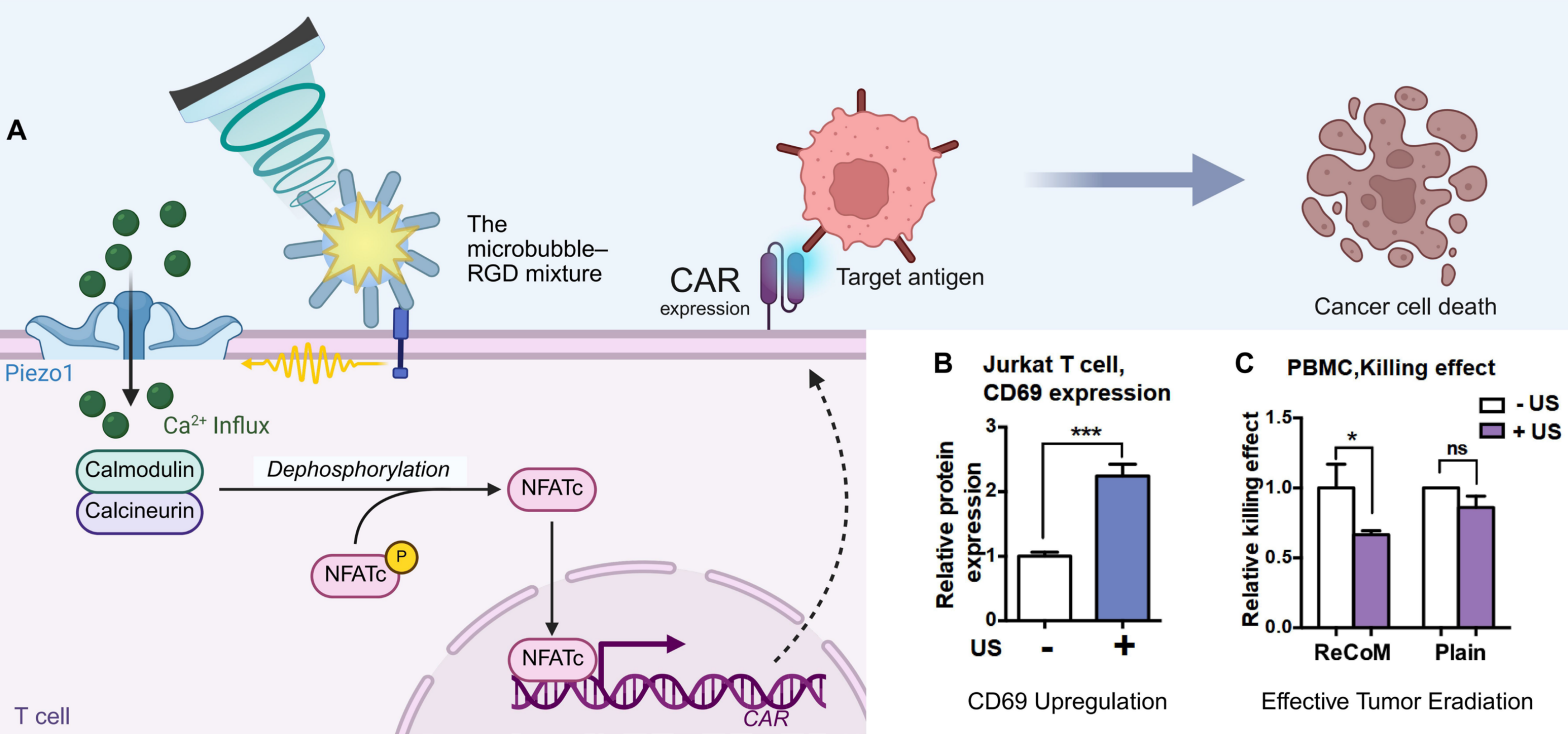
** Mechanogenetics for remote control of cancer immunotherapy. (A)** The schematic mechanism illustrates how sonogenetics induces the transcriptional expression of CAR. **(B)** The bar graphs show that ultrasound stimulation (+US) significantly upregulates the activation marker CD69 in Jurkat cells upon engagement with CD19⁺ tumor cells. **(C)** Luciferase-based killing assay shows that the ReCoM-engineered PBMCs demonstrate significant tumor killing efficacy upon ultrasound stimulation. Adapted with permission from 42, copyright 2018 National Academy of Sciences. Recreated with BioRender.com. CAR: chimeric antigen receptor; NFATc: nuclear factor of activated T-cells, cytoplasmic; PBMC: peripheral blood mononuclear cell; RGD: arginine-glycine-aspartic acid (peptide motif); US: ultrasound.

Current sonogenetic approaches in oncology can be categorized into three primary strategies. First, sonogenetics can directly suppress tumor growth. Piezo1 is highly expressed in the cell membrane of pancreatic cancer cell lines, particularly BxPC3. The activation of Piezo1, induced by external mechanical stimuli of ultrasound and MBs, mediates Ca²⁺ influx and mitochondrial dysfunction, ultimately leading to apoptosis in pancreatic cancer cells. In tumor xenograft models, US+MBs therapy significantly delayed tumor growth 146. Alternatively, ultrasound has been shown to suppress the growth of tumors established from A549 cells that exogenously express MscL-I92G/I96G 28. Second, sonogenetics can remotely control therapeutic immune cells. Ultrasound-activated Piezo1 in engineered T cells drives a calcium-dependent nuclear factor of activated T cells (NFAT) signaling pathway, which in turn induces the expression of chimeric antigen receptors (CARs) (**Figure 8A**). This enhances anti-tumor cytotoxicity in a spatiotemporally controlled manner (**Figure 8B, C**) 42. Third, a novel "Logic AND-Gated Sonogene Nanosystem" was devised to precisely regulate tumor cell apoptosis. In this framework, LIFU (0.25 MPa) activates MscL I92L expressed in tumor cells to induce Ca^2+^ influx. When intracellular Ca^2+^ was excessive, it triggered the Ca^2+^ apoptosis pathway of cells. Experiments involving multiple tumor cell lines (HeLa, B16, 4T1) as well as *in vivo* treatment of the B16 tumor models, have demonstrated favorable tumor-suppressing effects 87. The application of sonogenetics in the domain of tumor therapy has yielded a novel concept for precise, noninvasive eradication of tumor cells.

Similar to the synergy observed in the therapeutic application of the combination of sonogenetics and CAR-T, sonogenetics has the capacity to be integrated with conventional therapeutic modalities to confer spatiotemporal controllability. For example, combining sonogenetics with engineered bacteria allows for the localized, programmed release of drugs. Combining sonogenetics with systemic immune checkpoint inhibitors (e.g., anti-PD-1) is expected to overcome the immunosuppressive effect of the tumor microenvironment, enhancing the synergistic anti-tumor response. Drawing on the successful application of cell therapy in optogenetics 147, we propose to combine sonogenetics with stem cell technology: guiding embryonic stem cells (ESCs) or induced pluripotent stem cells (iPSCs) to differentiate into target cell types *in vitro* and undergo genetic engineering to stably express mechanosensitive proteins. Subsequently, these sonosensitive cells are transplanted into specific sites for targeted therapeutic intervention.

## 7. Conclusion and Future Outlook

Sonogenetics capitalizes on the comprehensive advantages of noninvasiveness, high spatiotemporal controllability, and deep tissue penetration. Compared to optogenetics, magnetogenetics, and chemogenetics (**Table 4**), it establishes a new paradigm for cellular regulation in precision medicine. This enables noninvasive treatment of deep-tissue diseases and mechanistic investigation, highlighting its irreplaceable value in precision medicine research 97,148. In particular, for neuromodulation and the treatment of deep tissues, the penetrating power of ultrasound is unrivaled by technologies such as optogenetics. For instance, in the management of refractory epilepsy, sonogenetics can modulate deep-brain circuits without the requirement for invasive fiber-optic implants 139, thereby avoiding risks of neuroinflammation and glial scarring. For deep visceral tumors such as pancreatic cancer, the superior penetration of ultrasound overcomes the severe light-scattering limitations of optogenetics 70. With the continuous development of portable ultrasound equipment, sonogenetics provides new methods for the long-term management of chronic diseases such as Parkinson's disease.

In order to facilitate the clinical translation of sonogenetics, there are several critical issues that must be addressed. The lack of a unified standard for frequency, intensity, and duty cycle hinders cross-study comparisons of distinct mechanosensitive transducers. Improper parameter settings can either reduce the regulatory efficiency or trigger adverse thermal effects and inertial cavitation, leading to biological tissue damage 157. Second, because few studies have investigated the distribution and responsiveness of endogenous MS ion channels across different tissues, activating exogenously expressed target MS ion channels may inadvertently affect non-target cells 158,159. Such off-target activation impairs the reliability of sonogenetics as a precision regulatory tool. Microbubbles designed to target specific cells at the molecular level 10,43 are expected to address this issue. Given the off-target effects of endogenous channels, we recommend prioritizing the selection of exogenous channels that offer high specificity for high-precision clinical applications. Furthermore, achieving bidirectional and precise control over cellular activity remains a significant challenge. To date, among the studies of MS channels, only the K2P channel family (such as TRAAK, TREK-1, TREK-2) exhibits K⁺ efflux upon activation, producing hyperpolarizing effect. The identification and introduction of additional inhibitory channels, such as K⁺ or Cl⁻ channels, are critical for expanding the sonogenetic toolkit. Additionally, as sonogenetics relies heavily on effective gene delivery, the inherent limitations of this delivery process pose a critical challenge. In practical clinical settings, we must consider potential pharmacological interference. Other drugs that the patient is taking, such as calcium channel blockers or neuromodulators, may inadvertently change the resting membrane potential or gate threshold of the target MS channels. Therefore, when selecting ultrasound parameters, the specific situation of the patient should be fully considered.

Sonogenetics is a multidisciplinary field. To promote the clinical translation of sonogenetics, interdisciplinary cooperation is important. Therefore, we present the following strategic roadmap organized by discipline. In molecular biology, we modify channels to achieve heightened ultrasonic sensitivity and optimize gene delivery protocols. Researchers should continue to explore novel MS ion channels and their mutants to improve mechanical sensitivity and achieve faster response speed. Furthermore, the biophysical properties of known MS ion channels, such as activation thresholds and biosafety, must be comprehensively characterized. Introduce certain well-researched MS ion channels, such as MscL-G22S, into non-human primates to further verify their safety and effectiveness under conditions closer to human environments. In engineering, we continuously refine the focusing precision of ultrasonic devices and develop lightweight and portable equipment for long-term management of chronic diseases. CNS represents a key application area of sonogenetics. Noninvasive ultrasound devices capable of penetrating the skull and delivering precise focusing in humans require further development in order to fully realize the benefits of sonogenetics in terms of precision and noninvasiveness. In clinical medicine, standardized clinical workflows must be established in accordance with FDA guidelines (MI, I_SPTA_). The determination of ultrasound parameters, imaging monitoring of MS ion channel expression and localization, and the establishment of standardized metrics should be integrated to form a closed-loop regulatory and efficacy assessment system. Moreover, doctors can design safe sonogenetic approaches tailored to disease characteristics. Finally, we can draw valuable experience from analogous technologies to advance sonogenetics. Future research should explore multimodal synergies to combine the strengths of different modalities. For instance, sono-optogenetics increases the penetration depth 160 and autophagy is regulated through combined optogenetics and chemogenetics 161. The construction of such a multimodal system may provide some means to overcome current technological limitations.

In essence, sonogenetics resolves the long-standing conflict between "precision" and "noninvasiveness" in precision medicine. It is expected to change the treatment pattern of incurable diseases such as neurodegenerative disorders, tumors, and visual impairments. Through systematic optimization tools, the establishment of standardized workflows, and strict clinical verification, sonogenetics will become a cornerstone of noninvasive precision medicine, bringing new hope to patients with unmet clinical needs currently.

## Figures and Tables

**Table 1:** Summary of research on mechanosensitive channels in sonogenetics.

Family	Mutations	Subjects (*in vitro/in vivo*)	Ion channel type	Ultrasonic response/pressure	Safety	Ref.
MscL	MscL-G22S	·HEK 293T cells and mouse primary neurons ·Neurons in the cerebral cortex or dorsomedial striatum of mice	Non-selective large-pore (>30 Å) ion channel, allowing passage of ions (including Ca^2+^) and small proteins.	500 kHz center frequency, 1 kHz PRF, 40% duty cycle	Expression of MscL-G22S shows no obvious side effects judged by mouse behavior, body weight, and resting membrane potential of neurons.	12
	MscL-I92G/I96G	·A549 cells ·mouse	Non-selective pore (30 Å in diameter) that allows easy passage of ions and small molecules below 1 kDa	0.044 MPa sound pressure (*in vitro*) 0.053 MPa sound pressure, equivalent to I_SPTA_ of 15.2 W/cm^2^ (*in vivo*)	/	28
	MscL I92L	·Rat hippocampal neurons in primary culture	Slightly more permeable to cations than anions	0.25 MPa peak negative pressure	/	27
TRP	TRP-4	·ASH, AWC, PVD sensory neurons and AIY interneurons of *C. elegans*	A stretch-sensitive, pore-forming mechanosensitive cation channel	2.25 MHz, 10-ms pulse, peak negative pressures of 0.41, 0.47, and 0.6 MPa (require gas-filled microbubbles)	Use low ultrasound peak negative pressures to avoid inertial cavitation.	9
	TRPP1/2 TRPC1	·Primary murine cortical neurons (endogenous expression)	Calcium-selective mechanosensitive ion channels	300 kHz, 15 W/cm^2^, 500 ms (CW)	No sustained calcium accumulation or irreversible membrane perforation was found after repetitive stimuli.	35
	hsTRPA1	·HEK 293T cells ·Mouse layer V motor cortical neurons (*in vivo*)	A widely conserved calcium-permeable nonselective cation channel	7 MHz transducer; 2.5 MPa (in* vitro*), 0.35-1.05 MPa (in* vivo*); 100 ms duration	hsTRPA1-expressing neurons could be repeatedly stimulated without apparent deleterious effects on cell health or a substantial decrement in calcium flux.	36,69
	TRPC6	·Cultured mouse cortical neurons and hippocampal neurons (endogenous expression)	A mechanosensitive nonselective cation channel	1 MHz, 190 mW/cm², 500 ms	/	37
Piezo	Piezo1	·HEK293T cells, Jurkat T cells, PBMCs	Nonselective cation channels with a modest preference for Ca^2+^ over Na^+^	2 MHz transducer, ∼0.6 MPa acoustic pressure (require gas-filled microbubbles)	Does not cause damage to target cells. Ultrasound induces minimal alteration to target cells.	44
Prestin	Prestin (N7T, N308S)	·SH-SY5Y cells ·C57BL/6J mice	Anion transporters	0.5 MHz, 0.5 MPa, 0.1% duty cycle, 3 s transient	Gene expression decreased markedly within 7 days *in vivo*.	51
	Prestin (N548S, V715G)	·HEK293T cells, HeLa cells, Primary neuron ·Hippocampal neurons and motor cortex of C57BL/6JNarl (B6) mice	Anion transporters	0.5 MHz, 0.5 MPa, 10 Hz PRF, 2000 cycles per burst, 3 s duration	No tissue disruption or hemorrhage are observed.	52
K2P	TRAAK	·*Xenopus laevis* oocytes	Mechanosensitive K+ channels	10 ms, 5 MHz, 1.2/3.6 W/cm^2^	The activation does not require cavitation or thermal effects, supporting the safety.	61
ASICs	ASIC1a	·CHO, Primary cortical neurons ·Cortex, hippocampus (CA1/CA2, dentate gyrus) and amygdala of C57BL/6J mice, Asic1⁻/⁻ mice, Asic3⁻/⁻ mice	A sodium channel, resulting in the intracellular calcium elevation possibly by activating voltage-gated calcium channels	1 MHz transducer; 7.4 mW/cm^2^ (I_SPTA_) at 700 mVpp (*in vitro*); 5 mW/cm^2^ (I_SPTA_) at 900 mVpp (*in vivo*)	Use ultrasound exposure below 10 mW/cm^2^ (I_SATA_) to ensure safe therapeutic applicability.	63
YAP	YAP	·Murine C2C12 mesenchymal precursors (endogenous expression)	Mechanosensitive transcriptional coactivator	3.6 MHz excitation frequency, 100 Hz PRF, 27.8% duty cycle and I_SATA_ = 44.5 mW/cm^2^	/	65
MEC	MEC-4 and MEC-6	*·C. elegans* (endogenous expression)	DEG/ENaC family mechanosensitive channels	SAW: 27.4 MHz frequency, 3 MPa acoustic pressure, 5 W electronic power.	/	68

CHO: the Chinese hamster ovary; PBMCs: peripheral blood mononuclear cells; PRF: pulse repetition frequency; I_SPTA_: spatial peak temporal average intensity; I_SATA_: spatial average temporal average intensity; SAW: Surface acoustic wave.

**Table 2:** Comparison of key characteristics, mechanisms, and properties of viral and non-viral gene delivery vectors.

	Core Components	Delivery mechanism	Gene capacity	Immunogenicity	Expression duration	Cell targeting	Ref.
AAVs	Target gene expression cassettes + inverted terminal repeats (ITRs) + capsid proteins (VP1/VP2/VP3)	The AAV vector undergoes receptor-mediated endocytosis, achieves endosomal escape, and translocates to the nucleus to facilitate gene expression.	~5 kb	Low, but faces the issue of pre-existing immunity against natural serotypes.	Transgene expression can endure for several years.	Serotypes naturally target different tissues.	94-96
LVs	Capsid proteins + unrelated virus + recombinant viral genome	Single-stranded RNA (ssRNA) is reverse-transcribed and semi-randomly integrated into the host genome.	~9kb	Concerns regarding immunogenicity exist for *in vivo* applications. Innate immunity is easily triggered but controllable; adaptive immunity is low risk but requires vigilance.	Long-term stable expression is achieved due to the integration of the gene into host cells, but it carries the risk of insertional mutagenesis.	Alter the envelope glycoproteins (pseudotyping) to regulate tropism.	97,98
Adv	The protein capsid encapsulating the genome	Double-stranded DNA (dsDNA) is delivered and persists as free entities within the nuclei of transduced cells.	~30kb	High	Medium-term expression	/	99-101
LNPs	Cationic or ionizable lipid, a helper lipid, a PEG-lipid, and cholesterol	Enter cells via endocytosis, then escape from the endosome.	Flexible	Significantly lower than viral vectors	Transient expression	Most intravenously administered nanoparticles accumulate in the liver.	102,103
MB-assisted US-guided gene delivery	Core (SF₆, C₃F₈, C₄F₁₀) + shell (polymers, proteins, surfactants, or phospholipids)	The gene is loaded into the MB shell layer. Ultrasound triggers the rupture of the MBs to release genes, synchronizing with the opening of the BBB.	Limited	Lower than viral vectors	Transient expression	Control of ultrasound irradiation area and modification of microbubbles	104-106

AAV: adeno-associated virus; ITRs: inverted terminal repeats; VP: viral protein; LV: lentivirus; Adv: adenovirus; LNP: lipid nanoparticle; PEG: polyethylene glycol; MB: microbubble; US: ultrasound; BBB: blood-brain barrier.

**Table 3:** Summary of therapeutic applications of sonogenetics in diverse disease models.

Disease Models	Targets	Transducers	Mechanism	Therapeutic efficacy	Ref.
Parkinson's disease	STN	MscL-G22S	MscL-G22S activation excites STN neurons, modulating the specific brain circuit.	Improvement in balance and motor endurance.	134
	Dopaminergic neurons of the SN	mPrestin (N7T, N308S)	Neuronal activation promotes expression of neurotrophic factors such as BDNF and NGF, thereby slowing the loss of TH+ neurons and restoring dopamine synthesis capacity.	After an 8-week course of treatment, dopaminergic neurodegeneration was reduced tenfold, and motor symptoms were alleviated fourfold.	132
Epilepsy	GABAergic neurons	mPrestin (N7T, N308S)	Expressed in GABAergic neurons, mPrestin can be activated by 0.5 MHz transcranial ultrasound, causing these neurons to release GABA, which inhibits the abnormal synchronous excitation of nerve cells.	Upon ultrasound stimulation, epileptiform abnormal activity is directly reduced by 50%, and this inhibitory effect persists for 60 minutes.	139
Reward-related behavior	VTA	MscL-G22S	Activation of MscL in VTA neurons leads to dopamine release in the NAc (mesolimbic pathway activation).	Induction of appetitive conditioned reflex behavior.	134
Depressive-like behavior	DRN neurons	Endogenous sonosensitive mediators	Ultrasound mechanical forces amplified by PGVs can activate these channels, leading to rapid release of 5-HT.	Significant reduction in immobility time and prolongation of struggle time (TST/FST).	10
Visual restoration	V1 cortex	MscL-G22S	Activation of MscL triggers the generation of electrical signals in visual pathway neurons. These signals are decoded and processed by higher-order visual areas to form visual perception.	For mice expressing MscL, ultrasonic stimulation shows no statistically significant difference in success rates from the light-stimulated control group in associative learning tasks.	6
Tumor Therapy	Pancreatic cancer cells	Endogenous Piezo1	Piezo1 is activated by external mechanical stimuli from US and MBs, mediating Ca²⁺ influx, inducing mitochondrial dysfunction, and ultimately leading to pancreatic cancer cell apoptosis.	Significantly delay tumor growth with US+MBs therapy.	146
	Tumor cells	MscL I92L	Delivery of MscL I92L to tumor cells via cationic nanoliposomes, followed by ultrasonic activation of the channel, induces excessive Ca²⁺ influx and precisely triggers tumor cell apoptosis.	In mouse tumor models, the system successfully inhibits tumor growth and significantly improves survival rates.	87
	T cells (Jurkat T-cell lines/PBMC)	Endogenous Piezo1	Piezo1 is activated by ultrasound + microbubbles, generating Ca²⁺ influx. Calcineurin, activated by Ca²⁺ influx, induces NFAT response elements, ultimately driving CAR gene expression.	Remote control of T-cell activation (CD69 upregulation) and CAR expression; enhanced cytotoxicity of ultrasound-induced PBMCs against target Nalm6 tumor cells.	42
	NSCLC A549 cells	MscL-I92G/I96G	Ultrasound activation of MscL induces calcium influx and cytoplasmic vacuolization, and decreases cell viability.	Reduced tumor volume and tumor weight.	28

STN: subthalamic nucleus; TH: tyrosine hydroxylase; GABA: gamma-aminobutyric acid; VTA: ventral tegmental area; NAc: nucleus accumbens; DRN: dorsal raphe nucleus; PGVs: PEGylated gas vesicles; 5-HT: 5-hydroxytryptamine; TST: tail suspension test; FST: forced swim test; V1: primary visual cortex; US: ultrasound; MB: microbubble; CAR: chimeric antigen receptor; NFAT: nuclear factor of activated T-cells; PBMC: peripheral blood mononuclear cell; NSCLC: non-small cell lung cancer.

**Table 4:** A comparative summary of sonogenetics, optogenetics, magnetogenetics, and chemogenetics.

Technology	Toolkit	Mechanisms	Advantages	Limitations	Applications	Ref.
Sonogenetics	MS ion channels; Gene delivery vectors; Ultrasound	US-mediated mechanical effects (activation of mechanosensitive ion channels or macromolecular systems) or thermal effects (activation of thermoproteins or thermoresponsive promoters via elevated temperatures) enable remote regulation of cellular activity.	·High spatial and temporal resolution; ·Noninvasive regulation	·Off-target effect; ·Ultrasound parameter settings	·Parkinson's disease ·Epilepsy; ·Neural circuits regulating; ·Visual restoration; ·Tumor therapy	3,149
Optogenetics	Photoresponsive proteins; Gene delivery vectors; Light delivery devices	Light delivery devices provide light of matching wavelength to activate photosensitive proteins. Upon receiving light signals, these photoreceptive proteins undergo conformational changes, triggering ion currents or protein interactions.	·High spatial and temporal resolution; ·Strong cell type specificity	·Low penetration depth; ·Invasive; ·Immune response and thermal damage from implanted devices	·Retinitis pigmentosa; ·Deafness; ·Epilepsy; ·Parkinson's disease; ·Motor recovery after injury; ·Chronic pain; ·Cardiac arrhythmias; ·Laryngeal paralysis; ·Pancreatic islet-like organoid	150,151
Magnetogenetics	MNPs; Magnetic field	This technology exploits MNPs to induce mechanical or thermal stimuli within cells, thereby activating mechanosensitive and thermosensitive proteins.	·Noninvasive regulation ·Multimodal	·Precision of regulation; ·Clinical safety; ·Equipment condition; ·Unclear mechanism	·Cancer therapy; ·Stem cell differentiation and tissue formation; ·Magnetically sensitive insulin-producing cells	152,153
Chemogenetics	DREADDs; CNO; AAV vectors	Through genetic engineering, receptors are designed to respond specifically to biologically inert ligands, thereby enabling remote regulation of cellular activity	·High specificity; ·Duration of action	·Low time accuracy; ·Side effects	·Obesity; ·Sleep disorders; ·Epilepsy	154-156

MS: mechanosensitive; US: ultrasound; AAV: adeno-associated virus; DREADDs: designer receptors exclusively activated by designer drugs; MNPs: magnetic nanoparticles; CNO: clozapine-N-oxide.

## Abbreviations

5-HT: 5-Hydroxytryptamine
AAV: adeno-associated virus
AdV: Adenovirus
ARF: acoustic radiation force
ARG: acoustic reporter gene
ASIC: acid-sensing ion channel
ATC: acoustic tweezing cytometry
BBB: blood-brain barrier
BDNF: brain-derived neurotrophic factor
BMI: brain-machine interface
BURST: burst ultrasound reconstructed with signal templates
CAR: chimeric antigen receptor
CNO: clozapine-N-oxide
CNS: central nervous system
DEG/ENaC: degenerin/epithelial Na^+^ channel
DREADDs: designer receptors exclusively activated by designer drugs
DRN: dorsal raphe nucleus
dSTR: dorsal striatum
ESC: embryonic stem cell
FUS: Focused Ultrasound
GABA: gamma-aminobutyric acid
GFP: green fluorescent protein
GV: Gas Vesicle
HEK 293T: human embryonic kidney 293T
hSyn: human synapsin
iPSC: Induced Pluripotent Stem Cell
I_SATA_: spatial average temporal average intensity
I_SPTA_: spatial peak temporal average intensity
K2P: two-pore domain potassium channel
LIFU: low-intensity focused ultrasound
LNP: lipid nanoparticle
LV: lentivirus
MB: microbubble
MNP: Magnetic Nanoparticle
MRI: magnetic resonance imaging
MRgFUS: magnetic resonance-guided focused ultrasound surgery
MS (channels): Mechanosensitive (channels)
MscL: large-conductance mechanosensitive channel
NAc: nucleus accumbens
NFAT: nuclear factor of activated T-cells
NGF: nerve growth factor
NSCLC: non-small cell lung cancer
PBMC: peripheral blood mononuclear cell
PD: Parkinson's Disease
PEG: Polyethylene Glycol
PGVs: PEGylated Gas Vesicles
PTMB: Piezo1-targeted microbubble
SAW: Surface acoustic wave
SN: substantia nigra
STN: subthalamic nucleus
tDCS: transcranial direct current stimulation
TMS: transcranial magnetic stimulation
UMGAAV: ultrasound and microbubble guided adenoassociated viral vector
US: Ultrasound
V1: primary visual cortex
VTA: ventral tegmental area
WT: wild-type
YAP: yes-associated protein
YFP: yellow fluorescent protein
